# Optimization of Insulation Structure Design for Enameled Wires Based on Molecular Structure Design

**DOI:** 10.3390/polym17081002

**Published:** 2025-04-08

**Authors:** Yang Yu, Siyuan Li, Ling Weng, Xiaorui Zhang, Laiweiqing Liu, Qingguo Chen

**Affiliations:** 1 Key Laboratory of Engineering Dielectrics and Its Application, Ministry of Education, Harbin University of Science and Technology, Harbin 150080, China; yyu@hrbust.edu.cn (Y.Y.); lisiyuanhrbust@163.com (S.L.); zxrhrbust@163.com (X.Z.); qgchen@263.net (Q.C.); 2 School of Electrical and Electronic Engineering, Harbin University of Science and Technology, Harbin 150080, China; 3 School of Materials Science and Chemical Engineering, Harbin University of Science and Technology, Harbin 150040, China

**Keywords:** enameled wire, design of insulation structures, electric stress control, polyimide

## Abstract

The performance of enameled wires has an important impact on new energy vehicle motors. The mainstream practice of existing technology is to improve partial discharge inception voltage (PDIV) by doping powder to inhibit corona and increase varnish thickness, the limitations of which are also obvious. Powder doping has the problem of dispersion stability, and increasing the varnish thickness affects the size and power density of the motor. In this paper, a novel insulation structure design was given. The electronic field stress was controlled by using different dielectric constant materials, and the dielectric constants can be controlled by adjusting the free volume of the polymer. Finally, we specifically create a preparation scheme to increase the corona voltage and the PDIV, without a loss of the breakdown margin of the enameled wire, and the simulation results show that the outermost electric field strength of the enameled wire model decreases by 22.11% and the enameled wire breakdown margin increases by 26.85%.

## 1. Introduction

The performance of motor winding has an important impact on the lifespan of motors in new energy vehicles. With the development of electromagnetic flat-wire drive motors for new energy vehicles, greater power density and higher electric field strength necessitate rapid updates, upgrades, and iterations of insulation materials [1,2]. Although the slot filling rate of flat-wire motor windings used in new energy vehicles has improved considerably compared to that of round-wire motors, there remains potential for further advancements in improving power density and reducing energy loss [3]. Additionally, the higher voltage levels and operating frequencies make the interturn and outlet ends of the motor windings more prone to corona discharge and insulation failure [4,5,6]. Therefore, improving motor processing efficiency and the slot filling rate at the same time enhances the motor winding corona resistance and ensures its insulation performance, which are crucial for developing new energy motor winding insulation materials and for improving motor drive capabilities [7,8,9].

There are two main research directions for improving the insulation performance of equipment [10]. One perspective is to increase the thickness of the insulation layer. Through Gauss’ law, when the thickness of the insulation layer increases, the external field strength will be reduced. First, this reduces the possibility of internal material breakdown; secondly, this decreases the degree of ionization of the air in its vicinity, achieving the ability to resist corona. Also, due to the increase in thickness, the partial pressure at the location prone to partial discharge is reduced, which improves the PDIV of the material. However, increasing the thickness of the insulation layer can cause the insulation system of the equipment to take up too much volume and thus affect the efficiency of the motor. The second perspective is the doping of nanoparticles into the insulating polymer structure, which has been the focus of many previous studies [11,12,13,14]. Many previous papers have investigated its complex mechanisms and concluded that the effect of doping inorganic powders on enhancing the insulating properties is remarkable [15,16,17]. For practical applications, however, there are several problems with the doping of inorganic powders. Firstly, for the polymerization process, during the solvent evaporation of the polymer solution, some of the nanoparticles will settle to the bottom of the polymer solution due to gravity, resulting in poor dispersion of the inorganic powder; secondly, complete uniformity of particle size is difficult to achieve. Both issues make the dispersion of material property data large and thus unfavorable for use in high-risk areas.

In this paper, a polyimide matrix with sufficient molecular weight was prepared by the one-step method using isocyanate and anhydride as monomers, and then partial branching units were introduced into the connected segments by isocyanate trimers. By using trimers as branching units, a series of micro-branch polyimide materials was prepared and characterized. The expected effect of changing the dielectric constant and increasing breakdown strength was achieved. This study provides the research idea of combining molecular structure design with insulation structure design.

## 2. Materials and Methods

### 2.1. Materials

The 3,3′,4,4′-Benzophenonetetracarboxylic dianhydride (BTDA) chemical was purchased from Tianjin Guangfu Fine Chemical Co., Ltd., Tianjin, China; the purity is 99% and was purified by sublimation prior to its use. Diphenylmethane diisocyanate (MDI-50), Hexamethylene Diisocyanate (HDI), and (2,4,6-trioxotriazine-1,3,5(2H,4H,6H)-triyl) tris(hexamethylene) isocyanate (HDI trimer) were supplied by Wanhua Chemical Group Co., Ltd., Yantai, China; the purity is 99.9%. N-methyl-2-pyrrolidone (NMP) was obtained from Tianjin Guangfu Fine Chemical Research Institute and treated with a 4 Å molecular sieve for more than 48 h for purification.

### 2.2. Preparation of BTDA/HDI/MDI/HDI Trimer–PI Films

**Scheme 1:**
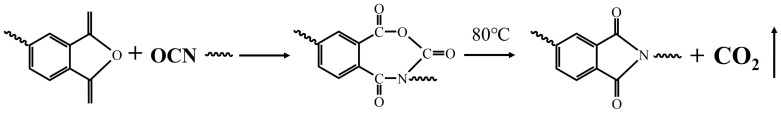
The reaction mechanism for the preparation of polyimides from anhydrides and isocyanates in a “one-step” process.

The isocyanate one-step method refers to the use of binary isocyanate and binary acid anhydride as reaction monomers, with the reaction conducted in a polar solvent by heating. The reaction mainly consists of two stages [18]. First, the anhydride group reacts with the isocyanate group (NCO-) to form an unstable seven-membered ring. The seven-membered ring decomposes into a five-membered imide ring at high temperatures and emits carbon dioxide gas. Compared with the traditional two-step method, this reaction does not generate intermediate polyamide acid (PAA) and does not require the process of thermoimidization, which makes the reaction temperature of the one-step reaction lower than that of the traditional two-step method. On the other hand, the reaction product is a direct polyimide solution, and the system is relatively stable and easy to preserve. The reaction mechanism for the preparation of polyimides from anhydrides and isocyanates in a “one-step” process is shown in Scheme 1. The one-step synthesis of polyimide is to weigh the raw material in a four-necked bottle according to a certain ratio, add MDI at the same time, control the reaction temperature, stir continuously during the reaction, and control it at a certain speed until the end of the reaction.

To a three-necked flask equipped with a mechanical stirrer, 3,3′,4,4′-Benzophenonetetracarboxylic dianhydride (BTDA, 10.0 mmol), (2,4,6-trioxotriazine-1,3,5 (2H,4H,6H)-triyl) tris (hexamethylene) isocyanate (HDI trimer, 0/0.1/0.2/0.3/0.4 mmol), and N-methyl-2-pyrrolidone (NMP, 22.5 mL) were added at 105 °C for 1 h. PI with a branched structure was obtained from polycondensation in a nitrogen atmosphere. Hexamethylene Diisocyanate (HDI, 3.0/2.7/2.4/2.1/1.8 mmol) and diphenylmethane diisocyanate (MDI-50, 7.0 mmol) were slowly added into the three-necked flask and reacted at 95 °C for 8 h. A series of PI solutions, each with a different multiple-branched cross-link structure, was obtained.

Following this, the PI solutions were cast onto dust-free glass and placed into a vacuum oven undergoing a programmed curing process at 80 °C for 2 h, 120 °C for 1 h, 160 °C for 1 h, and 220 °C for 2 h. Compared with the traditional thermal imidization process of PI films, the curing temperature was reduced. The flexible PI films with a thickness of 10~20 μm were obtained by immersing the glass in water and obtaining samples for measuring material properties.

### 2.3. Characterization

Fourier transform infrared spectroscopy (FTIR) was performed using the NICOLET iS10 infrared spectrometer manufactured by Thermo Fisher Scientific, Waltham, MA, USA. The spectral range was 4000~400 cm^−1^, with the highest resolution of 0.4 cm^−1^. The test resolution was 4 cm^−1^ with 16 scans. The solution of the test is 4 cm^−1^.

The relative permittivity and dielectric loss were measured on a precision impedance analyzer (German Alpha-A) with a platinum electrode (10 mm × 10 mm). The measurements were taken at room temperature with scan frequencies from 10 to 10^7^ Hz. The principle is to apply a frequency conversion AC signal (1V) on both sides of the sample, trigger the internal polarization behavior of the medium, record the current signal through the sample, and convert it into impedance. The capacitance C of the sample can be calculated using the following equation:

(1) $$C=\frac{1}{2\pi fX}$$

where *X* is the impedance of the sample and *f* is the test frequency. The relative dielectric constant of the sample can be obtained using the following formula:

(2) $$\varepsilon_{r}=\frac{C}{C_{0}}$$

where *C*_0_ is the capacitance when the electrode plate is in vacuum and *ε*_r_ is the capacitance gain multiple, that is, the relative dielectric constant. The loss factor (tan*δ*) parameter can be obtained by analyzing the phase difference between the sample voltage and current.

The breakdown field strength of PI films was measured with an HT-100 (Gljingu, Guilin, China) instrument in an oil bath environment, using a sample of size 100 mm × 100 mm × 20 μm and a ramp rate of 1000 V/s.

Flexural strength was carried out using an Instron universal testing machine at a speed of 10 mm/min. The specifications of the sample were 100 mm × 10 mm × 5 mm. The XJJ-5 impact testing machine from Chengde Testing Machine Co., Ltd., Chengde, China, was used for impact strength. Each sample was tested 10 times, the error point was removed, and the average values were taken. The dimension of the samples is 100 mm × 10 mm × 5 mm.

Dynamic mechanical analysis (DMA) scans were conducted with a TA Q800 (TA Instrument Company, New Castle, DE, USA) dynamic mechanical analyzer in stretching mode. The dimension of the sample was 40 mm × 0.9 mm × 7 mm at a heating rate of 3 °C/min at 1 Hz with a 50 μm amplitude in a range of 50–300 °C.

The thermal properties of the material were tested using a NETZSCH TG 209F3 thermogravimetric analyzer from NETZSCH Instruments, Munich, Germany. An analyzer with a N_2_ atmosphere, a mass of 10 mg of the test sample, a temperature range of 30 to 700 °C, and a temperature increase rate of 0.1 °C/min was used. The temperature range was 30–700 °C, the rate of increase was 10 °C/min, and the nitrogen flow rate was 20 mL/min.

## 3. Results and Discussion

### 3.1. Segment Structure Characterization and Analysis

**Figure 1:**
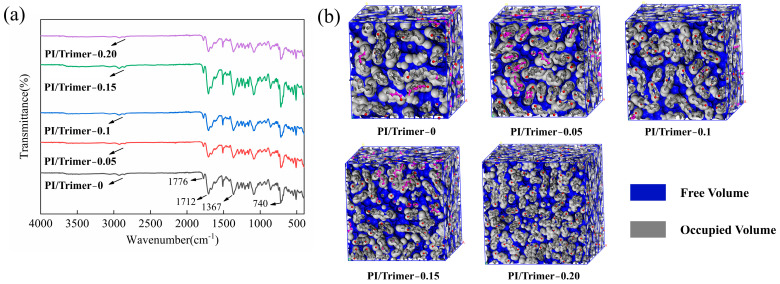
(**a**) PI–trimer films and their ATR-FTIR curves. (**b**) Simulation models of different HDI trimer contents.

FTIR curves of PI–trimer films are shown in Figure 1. The characteristic absorption peaks at 1776 cm^−1^ and 1712 cm^−1^ correspond to the asymmetric and symmetric stretching vibrations of the imine ring C=O. The peak at 1366 cm^−1^ is the stretching vibration peak of the imine ring C-N, and the peak at 740 cm^−1^ is the bending vibration peak of the imine ring. The presence of these absorption peaks is evidence of PI production. The absence of the characteristic C=N IR peak of isocyanate at 2270 cm^−1^ indicates that the reaction of isocyanate is complete.

The free volume is the sum of static voids created by chain packing and transient gaps created by thermally induced chain rearrangement. It should be noted that the transient gaps provide a path of low resistance for diffusing molecules [19]. The FFV of PI has been studied using both molecular dynamics simulations and experimental approaches to explore the relationship between the dielectric constant and the micro-branched structure. In our study, it was found that higher FFV was induced in PI films by incorporating HDI trimers. The variation in film density offered an explanation for the variations in the free volume and dielectric constant observed in the film. Figure 1b shows the schematic representation for a simulated molecular cell for all the samples, where the blue and gray areas represent the free volume and the occupied volume in BTDA/HDI/MDI/HDI trimer–PI, respectively. The FFV of each BTDA/HDI/MDI/HDI trimer–PI sample was calculated from the specific volume and occupied volume of the polymers based on their optimized model structure as follows:

(3) $$FFV=\frac{V-V_{0}}{V}=\frac{V-1.3V_{W}}{V}$$

where *V*, *V*_0_, and *V*_w_ are the specific volume, occupied volume, and van der Waals volume of the polymer, respectively.

The theoretical density, specific volume, and occupied volume were obtained from molecular dynamics simulation, and the final FFVs for all samples are summarized in Table 1. The density of PI in the stable state varied between 1.347 and 1.369 g/cm^3^. From the data in Table 1, it can be concluded that when the linear polyimide starts to introduce trimer branched units, the addition of trimers gradually breaks the regular arrangement between the chain segments, which enlarges the gaps between the linked segments, leading to an increase in the free volume of the material; however, as the trimer content further increases, the scale of the chain segments becomes progressively shorter, increasing the mobility of the chain segments, which in turn causes the arrangement of the chain segments to become more regular, resulting in a decrease in the free volume of the material.

### 3.2. Dielectric Properties

**Figure 2:**
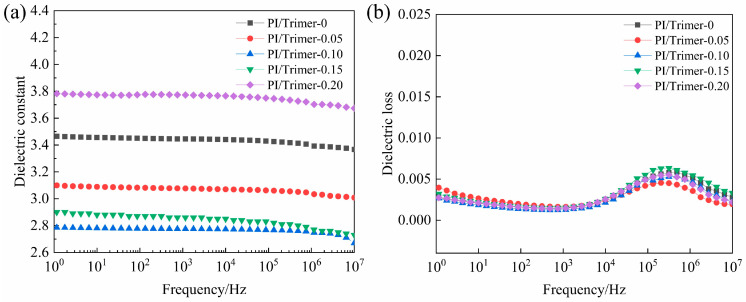
Dielectric constant and dielectric loss of different contents of HDI trimers: (**a**) dielectric constant and (**b**) dielectric loss.

Figure 2a illustrates the variations and differences in the dielectric constants (εr) of the BTDA/HDI/MDI/HDI trimer–PI films across the entire measurement frequency range. It is observed that the dielectric constants of all samples remain relatively stable in the low-frequency range, exhibiting a gentle decreasing trend as the frequency increases. The graph indicates that the dielectric constant of the material initially decreases and then increases with the rising trimer content. At a trimer concentration of 0.10, the dielectric constant of the film is measured at 2.8, which is 17.6% lower than that of the film without trimers. This reduction can be partly attributed to the free volume introduced by the HDI trimers, which significantly decreases the density of the polar groups, leading to a lower dielectric constant. In addition, the addition of the trimer results in the appearance of a micro-branched structure, which breaks the regularity among the chain segments and affects the crystallization of the polymer chain segments. While the dielectric constant was reduced to 2.8 by adding PI/trimer-0.10 HDI trimer, an excess of HDI trimer beyond PI/trimer-0.15 could cause it to increase again. This occurs because a higher HDI trimer content may gradually decrease the free volume by shortening the distance between molecular chains through the cross-linking effect [20,21]. When cross-linking becomes the dominant factor, the impact of the micro-branched structure on enhancing free volume is significantly diminished, resulting in an overall performance characterized by reduced free volume and increased *ε*_r_.

The dielectric loss characteristics of the samples are depicted in Figure 2b. In the frequency range of 10^5^–10^6^ Hz, the samples exhibit pronounced dielectric loss values due to dynamic polarization effects. This phenomenon occurs when the material’s polarization response lags behind the rapidly alternating electric field at elevated frequencies. During this intermediate frequency regime, the electric field performs substantial work on molecular dipoles, inducing rotational motion that manifests as increased energy dissipation. However, as the frequency exceeds 10^6^ Hz, the dipole polarization mechanism becomes effectively frozen as the polarization process cannot follow the ultrafast electric field variations, resulting in a marked reduction in dielectric loss. Notably, the dielectric loss tangent (tan *δ*) maintains remarkable stability across different HDI trimer concentrations, with all measured values consistently below 0.008 throughout the tested frequency spectrum. This exceptional performance stability, coupled with the low absolute loss values, satisfies the stringent requirements for advanced enameled wire applications where minimal dielectric dissipation is critical.

### 3.3. Insulation Properties

The breakdown field strength of materials is usually described through the Weibull cumulative distribution function with two parameters, using the following formula [22,23]:

(4) $$P(E)=1-\exp\left[-{\left(\frac{E}{E_{0}}\right)}^{\beta }\right]$$

Among them, *P*(*E*) is the cumulative failure probability; *E* is the breakdown strength; *β* is the shape parameter; and *E*_0_ is the breakdown field strength at *P*(*E*) = 63.28%. After taking the logarithm of both sides of the public display 4, the linear equation formula can be obtained as follows:

(5) $$\ln\left[-\ln(1-p)\right]=\beta (\ln E-\ln E_{0})$$

For each corresponding field strength *E*_0_, a *p*-value could be calculated, which is calculated using Formula (5), where *i* indicates that *E* values are arranged from small to large and n indicates the number of tests of each sample.

(6) $$p=\frac{i-0.5}{n+0.25}$$

**Figure 3:**
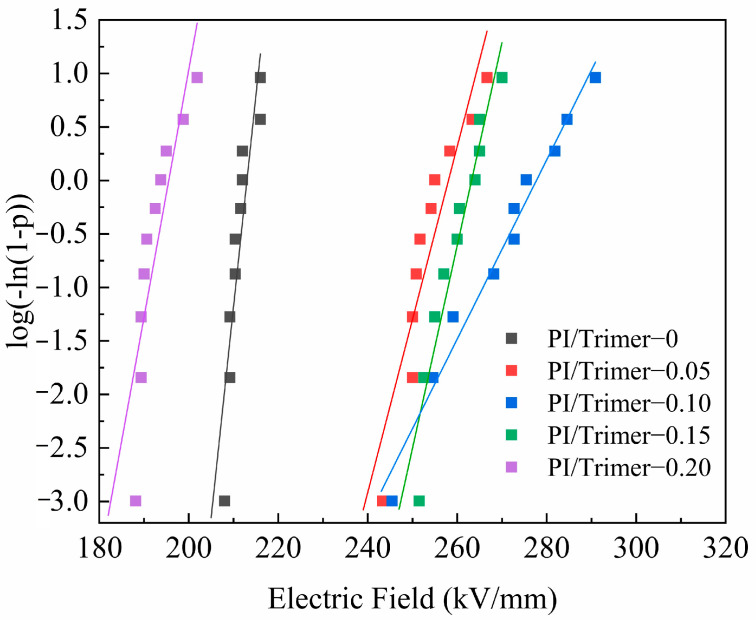
The breakdown field strength of the HDI trimer.

Through the dielectric breakdown test, the relationship between ln(−ln(1 − *P*(*E*))) and *E* is shown in Figure 3, which is a comparison of the breakdown field strengths of modified PI created with different HDI trimer contents. The breakdown field strength of the material increases with increasing trimer content when trimers are added in amounts up to 0.10 mmol. At a trimer addition of 0.10, the dielectric strength of the coating reached 270.1 kV·mm^−1^, an increase of 29.1% compared to the polyimide film without trimers. This is due to the addition of isocyanate trimers: the internal formation of a cross-linked network structure limits the orientation of the molecular chain segments under the action of the electric field, such that the defects that form when the material is subjected to electron impact cannot grow in the direction of the chain segment, so the breakdown performance of the insulating paint is improved. If too many branched structures are introduced, the structure between the molecules is too complex, making it difficult to form forces between the chain segments; as a result, electrons can easily pass through the gaps between the chain segments and eventually through the material to cause the body to break down, resulting in a reduction in the breakdown field strength of the material.

### 3.4. Insulation Structure Design

**Figure 4:**
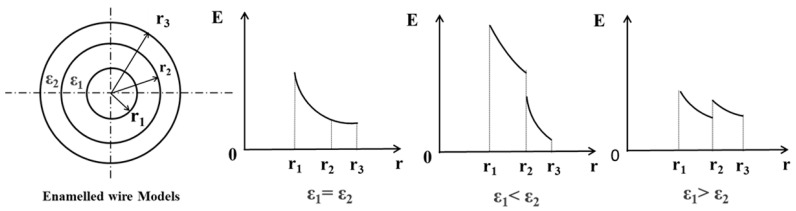
Principle of electric field regulation.

First of all, the design of the electric field strength at the outermost part of the insulation serves as a starting point, and through Gauss’ theorem, the voltage allocated to the material in an alternating current is related to the dielectric constant of the material. If we increase the dielectric constant of the material outer layer of an enameled wire, the electric field strength of the outer layer will decrease (*ε*_1_ < *ε*_2_ in Figure 4). This will reduce the degree of ionization of the surrounding air, thereby providing corona resistance. However, this method will increase the field strength within the insulation and thus increase the risk of breakdown within the insulation. For this problem, when the dielectric constants of the insulating layers are the same, the field strength inside the insulating layer is higher than the outer layer field strength, so the performance of the outer layer of the material is not fully utilized. Therefore, the previously used method of enhancing corona resistance involves gradually increasing the dielectric constant of the inner insulation layer from the inside to the outside. This approach aims to achieve a uniform electric field within the material, thereby reducing the likelihood of breakdown (*ε*_1_ > *ε*_2_ in Figure 4). The breakdown margin of the internal insulation layer is increased.

For improving the partial discharge starting voltage of the material, there are two perspectives: The first is for between the motor shell and the enameled line, which reduces the enameled line outside the electric field strength and the enameled line outside the air ionization degree, so as to achieve the effect of improving the enameled line corona voltage. The second is for the enameled wire turns, which reduce the external air part of the partial pressure, so that the air part of the voltage is lower than the air breakdown voltage as stipulated by Paschen’s law. Both directions are mentioned below.

So far, through designing the insulation structure of the wire, the problem of improving the insulation ability of enameled wire is transformed into the problem of how to control the dielectric constant of the material. To do so, many studies have been carried out, where the principle is mainly to change the number of dipoles per unit volume or to change the atomic structure of the polymer chain. Branched polymers are a way to change the dielectric constant of the polymer, and such polymer membranes are also widely used in membrane materials such as gas separation membranes and proton exchange membranes. Introducing a multiply branched structure into a linear polymer changes the free volume of the material, which in turn changes the number of polarized molecules per unit volume of the polymer, changing the dielectric constant of the material.

### 3.5. Electric Field Simulation of the Insulation Structure

Based on the previously mentioned design principles, the material with the highest dielectric constant should be used as the outermost layer of the enamel wire. To reduce the electric field in the interlayer, materials with varying dielectric constants are incorporated to create a more uniform electric field. Finally, to enhance the partial discharge inception voltage (PDIV), materials with lower dielectric constants are utilized whenever possible.

Special emphasis needs to be placed on the fact that it is common to measure the risk of breakdown of an enameled wire by the value of the breakdown margin (the multiplication of the breakdown field strength of a material relative to the field strength of the material at the time of use). For an enameled wire with the same dielectric constant, the field strength close to the conductor is greater than the field strength outside, which does not take full advantage of the properties of the material outside the insulation. To enhance the partial pressure of the outer material, it is essential to ensure that the total electric field strength of the insulation layer is effectively distributed. This approach reduces the pressure on the inner material, thereby allowing the material to perform at its full potential and ultimately improving the overall breakdown field strength of the enameled wire. However, in the series of polyimide materials examined in this study, each material exhibits varying breakdown field strengths. It is crucial to consider not only the distribution of electric field strength across each layer of the enameled wire but also the breakdown field strength of each individual layer to assess the breakdown margin of the enameled wire.

In this study, there are two limiting factors for improving the breakdown margin: The first is that the performance of a certain material layer itself is insufficient; the solution is to discard the material layer and use a material with a higher breakdown field instead. The second is that a certain material layer in the electric field of the partial pressure is too large, and the thickness of the material is too thin, resulting in a larger field strength, and the material performance can no longer be improved, but increasing the thickness of the layer could reduce the field strength and improve the breakdown margin.

**Figure 5:**
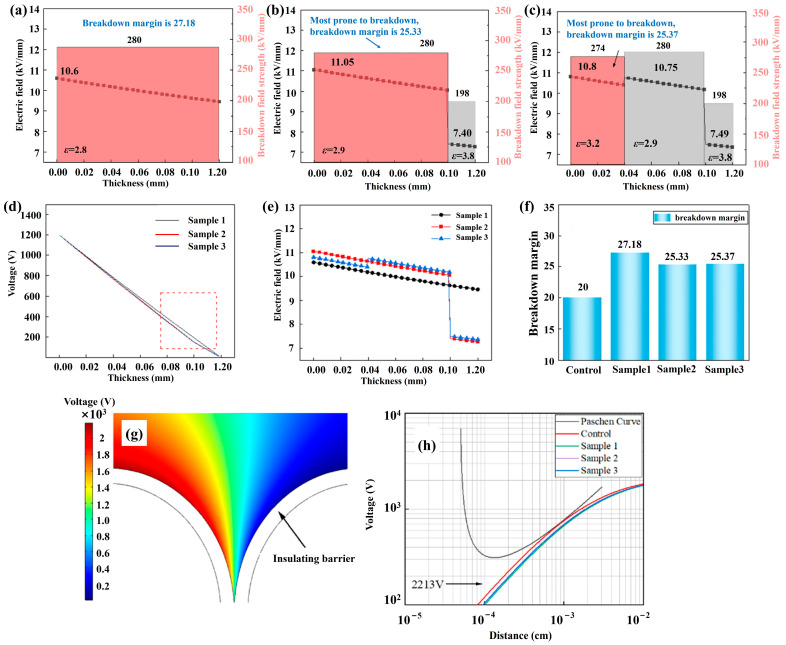
(**a**) The electric field strength at each position of Sample 1 and the breakdown margin of the insulating material at that position. (**b**) The electric field strength at each position of Sample 2 and the breakdown margin of the insulating material at that position. (**c**) The electric field strength at each position of Sample 3 and the breakdown margin of the insulating material at that position. (**d**) Voltage distribution for different regulation methods. (**e**) Field strength distribution for different regulation methods. (**f**) Comparison of breakdown margins by sample. (**g**) Calculation model for PDIV. (**h**) Corresponding voltage drop for different regulation methods at 2481V vs. corrected Paschen curve.

Based on the above analyses, three models of enameled wire with different dielectric structures are proposed, which represent the complete use of the material with the lowest dielectric constant and the highest breakdown field strength (Sample 1); an attempt to reduce the strength of the outermost electric field (Sample 2); and the reduction in the strength of the outermost electric field while taking into account the margin of the internal breakdown (Sample 3); these samples are compared with the completely unbranched and structured polyimide enameled wire (Control Group). The simulation data and comparison with polyimide enameled wires (Control Group), which have not been branched or structured at all, are in Figure 5. And the data of these enameled wire models are in Table 2.

As shown in Figure 5, when only the outer layer of the enameled wire is modified, the external field strength of the enameled wire is significantly reduced, but the internal field strength is significantly increased, and when the electric field is homogenized on the inside, the internal breakdown margin is increased, but due to the use of the trimer modification, the performance of the material itself is better than that before the modification. Therefore, no matter the design ideas, the breakdown margin and corona resistance of the enameled wire are improved.

### 3.6. Realistic Enameled Wire Production Process and Design Model Realization Methods

**Figure 6:**
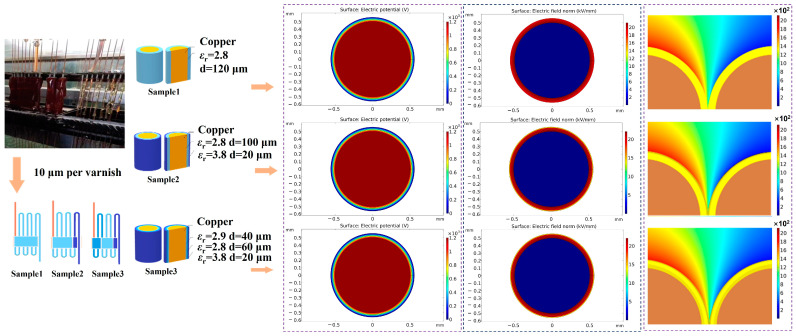
Comparison of the envisaged production process of magnet wires and their specific simulation diagrams.

Figure 6 shows the proposed preparation process of the three models based on the actual production process of enameled wires, as well as the potential change, electric field distribution, and partial discharge model simulation diagrams between turns for the three models. From the diagram, it can be seen that the wire is produced from copper wire by means of a polymer solution. The polymer solution covers the copper wire after it passes through the enameled box with the polymer, after which the solvent in the solution is evaporated through a drying channel with high temperature, thus adhering the polymer to the surface of the copper wire. It is worth noting that during this process, the copper wire is often attached to only 5–10 μm of polymer material at a time, and the process is repeated several times, thereby making the solenoid wire closer to the required size. This is due to a single attached too-thick polymer solution, which will lead to the solvent in the evaporation process of the external solution volatilization first affecting the internal solvent evaporation and subsequently being close to the copper wire part of the solution in the solvent volatilization, which will be left in the outer layer of the channel, resulting in the entire solenoid wire being inside the large number of air holes that emerged. This is very fatal for the partial discharge of the enameled wire and the impact of the breakdown margin, so in practice, it is often necessary to use multiple layers of paint to minimize internal defects. In practice, due to the size of the equipment, the copper wire often passes through several identical enameled boxes, thus completing the entire production process of the enameled wire. We take this as a foothold and consider the study to be of greater practicality.

## 4. Conclusions

In this study, we propose a combination of molecular structure design and insulation structure design, as well as a preparation strategy for the design and preparation of enameled wires. Tests on polyimide films show that by changing the proportion of the branched structure within the polymer, the dielectric constant and breakdown field strength of the material can be changed to a certain extent, where the dielectric constant of PI/trimer-0.10 is 2.8 and the breakdown field strength is 280 kV/mm. PI/trimer-0.10 has a dielectric constant of 3.8 and a breakdown strength of 190 kV/mm. On the other hand, the simulation results show that the outermost electric field strength of the enameled wire decreases by 22.11%, the breakdown margin increases by 26.85%, and the turn-to-turn partial discharge initiation voltage increases by 8.90%, due to the combined effect of the material and insulation structure. This study suggests that the design concept through the combination of molecular structure design and insulation structure design may be a more effective solution to improve the voltage platform of electrical devices.

## Figures and Tables

**Table 1:** The theoretical density, specific volume, and occupied volume of simulation cells and FFV calculated for BTDA/HDI/MDI/HDI trimer–PI samples.

Sample	Density (g/cm^3^)	Specific Volume (Å)	Occupied Volume (Å)	FFV
PI/trimer-0	1.369	113,098.2	76,819.76	0.3207
PI/trimer-0.05	1.356	65,111.07	43,787.38	0.3274
PI/trimer-0.10	1.353	39,217.56	26,347.85	0.3282
PI/trimer-0.15	1.347	27,893.85	18,649.97	0.3314
PI/trimer-0.20	1.355	22,874.09	15,398.01	0.3268

**Table 2:** Outermost electric field strength, maximum electric field strength, PDIV, breakdown field strength of the material at the most vulnerable point of penetration (BMMP), and breakdown field strength margin for all modulation methods.

Sample	Control Group	Sample 1	Sample 2	Sample 3
Maximum field strength	10.60 kV/mm	10.60 kV/mm	11.05 kV/mm	10.80 kV/mm
Outermost field strength	9.45 kV/mm	9.45 kV/mm	7.27 kV/mm	7.36 kV/mm
PDIV	2213 V	2491 V	2435 V	2410 V
BMMP	212 kV/mm	280 kV/mm	280 kV/mm	274 kV/mm
Margin multiplier	20.00	27.18	25.33	25.37

## Data Availability

The original contributions presented in this study are included in the article/Supplementary Materials. Further inquiries can be directed to the corresponding author.

## Supplementary Materials

The following supporting information can be downloaded at: https://www.mdpi.com/article/10.3390/polym17081002/s1. The characterization and properties of the materials in this study can be found in the Supplementary Materials, which include chemical structure of PI films modified with HDI trimer, mechanic properties, thermomechanical analyzer and thermal stability analysis. Figure S1. Tensile strength, elongation at break, and tensile modulus of different content of HDI Trimer. Figure S2. DMA curve of PI films modified with HDI trimer. Figure S3. TGA curve of PI films modified with HDI trimer.

## References

[1] Vershinin A.V., Dragomirov M.S., Zaitsev A.M., Kruglikov O.V. (2008). Development of embodiments of variable-frequency induction motors. Russ. Electr. Eng..

[2] Qin Y., Zhang S.D., Han S., Xu T.T., Liu C., Xi M., Yu X.L., Li N., Wang Z.Y. (2021). Voltage-Stabilizer-Grafted SiO_2_ Increases the Breakdown Voltage of the Cycloaliphatic Epoxy Resin. ACS Omega.

[3] Vu V.B., Ramezani A., Triviño A., González-González J.M., Kadandani N.B., Dahidah M., Aguado J. (2022). Operation of inductive charging systems under misalignment conditions: A review for electric vehicles. IEEE Trans. Transp. Electrif..

[4] Yadav K.S., Sarathi R. (2015). Influence of thermally aged barrier on corona discharge activity in transformer oil under AC voltages. IEEE Trans. Dielectr. Electr. Insul..

[5] Janaj A., Bandi A., Desur P., Prabhu D., Datar P., Kulkarni R.M. (2023). Improving electric field distribution on corona ring by nano-coating. AIP Conf. Proc..

[6] Liu X., Liu P., Zhang T., Yuan P., Zhu H. (2020). Detection method of inter-turn insulation defects of inverter-fed motors under induced impulse voltage. IEEE Electr. Insul. Mag..

[7] Fallah-Arani H., Tehrani F.S., Koohani H., Elmdoust B., Nodoushan N.J., Shafiei Z. (2023). Optimization of resin content to improve electrical and mechanical properties of polymer-concrete line-post insulators used in electrical distribution networks. Electr. Power Syst. Res..

[8] Zhang C., Liu Z., Tang C., Zhang T., Zhang Y., Zhang Y., Chi Q. (2024). Study of the Dielectric and Corona Resistance Properties of PI Films Modified with Fluorene Moiety/Aluminum Sec-Butoxide. Polymers.

[9] Dong Z., He Q., Shen D., Gong Z., Zhang D., Zhang W., Jiang Y. (2023). Microfabrication of functional polyimide films and microstructures for flexible MEMS applications. Microsyst. Nanoeng..

[10] Bian X., Yu D., Meng X., MacAlpine M., Wang L., Guan Z., Yao W., Zhao S. (2011). Corona-generated space charge effects on electric field distribution for an indoor corona cage and a monopolar test line. IEEE Trans. Dielectr. Electr. Insul..

[11] Wang Z.Y., Sun X., Wang Y., Liu J.D., Zhang C., Zhao Z.B., Du X.Y. (2022). Fabrication of high-performance thermally conductive and electrically insulating polymer composites with siloxane/multi-walled carbon nanotube core-shell hybrids at low filler content. Polymer.

[12] Kwon T., Lee S.H., Kim J.H., Hong S.K., Kim M., Kim M., Kim D.K., Kim I.J., Song J., Lee D.H. (2023). Polypropylene nanocomposites doped with carbon nanohorns for high-voltage power cable insulation applications. Adv. Compos. Hybrid Mater..

[13] Huang X.Y., Thomas A. (2020). Nanodielectrics. IEEE Trans. Dielectr. Electr. Insul..

[14] Li Z., Okamoto K., Ohki Y., Tanaka T. (2011). The role of nano and micro particles on partial discharge and breakdown strength in epoxy composites. IEEE Trans. Dielectr. Electr. Insul..

[15] Zhou H., Lei H., Wang J., Qi S., Tian G., Wu D. (2019). Breaking the mutual restraint between low permittivity and low thermal expansion in polyimide films via a branched crosslink structure. Polymer.

[16] Sun Q., Feng Y., Guo J., Wang C. (2023). Achieving both low thermal expansion and low birefringence for polyimides by regulating chain structures. Eur. Polym. J..

[17] Liu Y., Zhang Y., Lan Q., Liu S., Qin Z., Chen L., Zhao C., Chi Z., Xu J., Economy J. (2012). High-performance functional polyimides containing rigid nonplanar conjugated triphenylethylene moieties. Chem. Mater..

[18] Chern Y.T., Tsai J.Y. (2008). Low dielectric constant and high organosolubility of novel polyimide derived from unsymmetric 1, 4-bis (4-aminophenoxy)-2, 6-di-tert-butylbenzene. Macromolecules.

[19] Yamanaka K., Jikei M., Kakimoto M.A. (2001). Preparation of hyperbranched aromatic polyimide without linear units by end-capping reaction. Macromolecules.

[20] Shiravand F., Ascione L., Persico P., Carfagna C., Brocks T., Cioffi M.O.H., Puglisi C., Samperi F., Ambrogi V. (2016). A novel hybrid linear–hyperbranched poly (butylene adipate) copolymer as an epoxy resin modifier with toughening effect. Polym. Int..

[21] Xu P., Du X., Cong P., Zhou Z. (2022). Properties of paving epoxy asphalt with epoxy-terminated hyperbranched polyester. Road Mater. Pavement Des..

[22] Yue D., Feng Y., Liu X.X., Yin J.H., Zhang W.C., Guo H., Su B., Lei Q.Q. (2022). Prediction of energy storageperformance in polymer composites using high-throughput Stochastic breakdownsimulation and machine learning. Adv. Sci..

[23] Li Y., Yin J., Feng Y., Li J., Zhao H., Zhu C., Yue D., Liu Y., Su B., Liu X. (2022). Metal-organic Framework/Polyimidecomposite with enhanced breakdown strength for flexible capacitor. Chem. Eng. J..

