# Supplementary material for: Optimization of Insulation Structure Design for Enameled Wires Based on Molecular Structure Design
Source: Polymers (Basel). 2025 Apr 8;17(8):1002. doi: 10.3390/polym17081002 (PMC12030633; doi:10.3390/polym17081002)
Supplement: Supplementary file 1 [file polymers-17-01002-s001.zip › polymers-3556283-supplementary.pdf]

Optimization of Insulation Structure Design for Enameled Wires Based on Molecular Structure Design

Yang Yu [1,2], Siyuan Li [1,2], Ling Weng [1,3,*], Xiaorui Zhang [1,3], Laiweiqing Liu [3] and Qingguo Chen [1,2]

[1] Key Laboratory of Engineering Dielectrics and Its Application, Ministry of Education, Harbin University of Science and Technology, Harbin 150080, China; yyu@hrbust.edu.cn (Y.Y.); lisiyuanhrbust@163.com (S.L.); zxrhrbust@163.com (X.Z.); qgchen@263.net (Q.C.)
[2] School of Electrical and Electronic Engineering, Harbin University of Science and Technology, Harbin 150080, China
[3] School of Materials Science and Chemical Engineering, Harbin University of Science and Technology, Harbin 150040, China; liulai714@gmai.com
* Correspondence: l.weng@hrbust.edu.cn; Tel.: +86-0451-86392533

1. *Mechanic Properties*

The tensile strength, Young's modulus and elongation at break of BTDA/HDI/MDI/HDI Trimer-PI films are shown in Figure S1. The films have good mechanical properties with tensile strength of 296 MPa, Young's modulus of 6.25 GPa and elongation at break of 5.64%. The tensile strength and modulus were improved after the addition of 3% HDI trimer compared to the pure BTDA/HDI/MDI-PI films, indicating that HDI trimer has a greater effect on the tensile properties of the films. The tensile strength of the film increases significantly with increasing HDI trimer content, reaching a maximum at 3%, and begins to decrease above 5%. When HDI trimers are added to the system, their branched structure leads to localised cyclization of the linear polymer. If the branching density is not high, the cyclized structure will intersperse with other chain segments to form a cross-linked and interpenetrating network, resulting in greater force between chain segments and an increase in tensile strength; If the branching density is too high, the size of the cyclized structure becomes smaller and the chain segments are less likely to interpenetrate. In this case, the original regularity of the polymer chain segments is destroyed and the structure of the chain segments becomes loose and less interlocked. This is due to the fact that when the branched unit is introduced, the interpenetrating network structure restricts the movement of the chain segments, reducing the mobility and elasticity of the polymer chain segments and making it more difficult for the conformation of the chain segments to change when subjected to Stress.

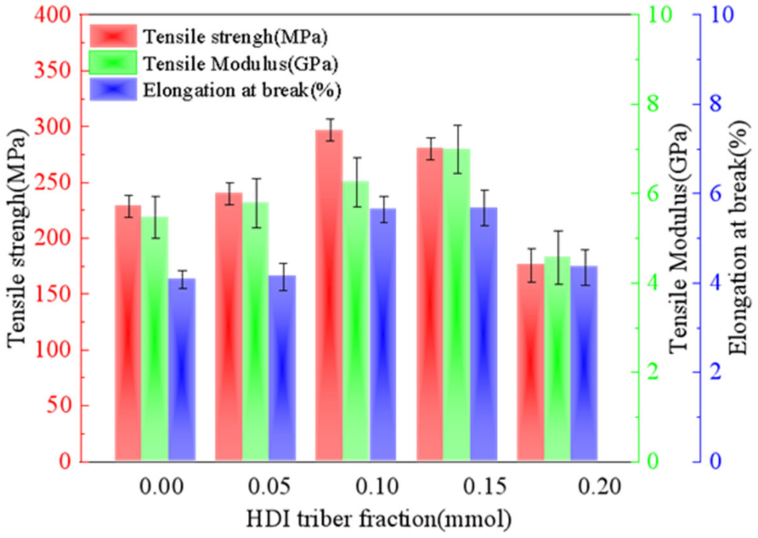

Figure S1. Tensile strength, elongation at break, and tensile modulus of different content of HDI Trimer.

2. Thermomechanical Analyzer

Figure S2 shows the DMA properties of materials with different trimer contents. Compared to pure linear PI films, the glass transition temperature of the material decreases slightly with the addition of small amounts of trimers. Flory's free volume theory suggests that a decrease in the Tg of the polymer occurs as the free volume increases. The increase in free volume gives the polymer chain segments room to move, resulting in a decrease in the glass transition temperature of the material, but as the trimer content increases further, the intertwining of the chain segments increases, limiting the mobility of the chain segments, causing the chain segments to be bound by other polymer segments, reducing their ability to move even though they have room to move freely, causing the Tg of the polymer to instead begin to increase.

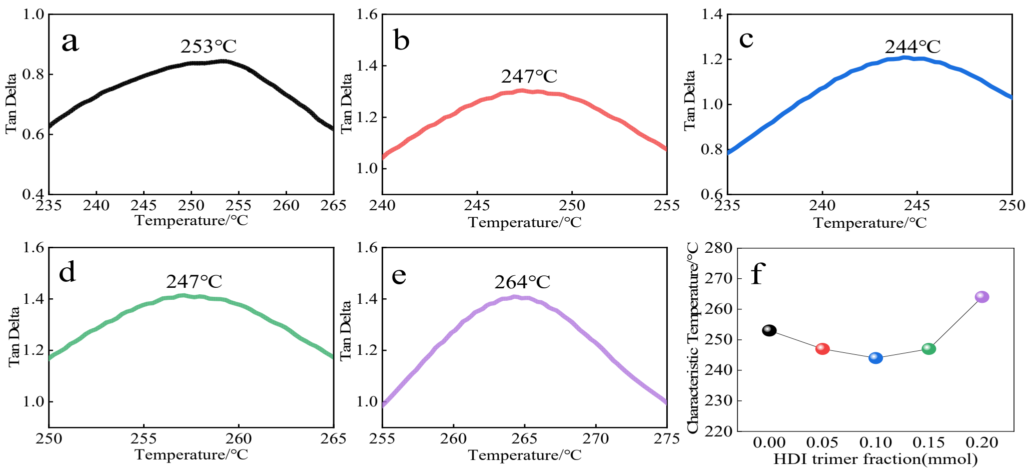

Figure S2. DMA curve of PI films modified with HDI trimer. a peak-sharp diagram of pure PI films; b peak-sharp diagram of HDI trimer (the content was 0.05 mmol); c peak-sharp diagram of HDI trimer (the content was 0.10 mmol); d peak-sharp diagram of HDI trimer (the content was 0.15 mmol); e peak-sharp diagram of HDI trimer (the content was 0.20 mmol).

3. *Thermal Stability Analysis*

The heat resistance of the material is shown in figure S3, with thermal decomposition of the material starting at around 400 °C. The main problem is that the overall heat resistance of the material is affected by the low heat resistance temperature of the long methylene chains introduced by the HDI. On the other hand, it can be seen from the graph that the heat resistance of the material decreases slightly as the trimer content increases. This is due to the fact that as the trimer content increases during the synthesis process, the viscosity of the reaction system increases significantly, leading to difficulties in the movement of the chain segments in the later stages of the reaction, which in turn leads to a reduced probability of collision of the reacting groups and affects the degree of reaction, resulting in the end groups containing unreacted groups and the end groups being less heat resistant than the molecular chains themselves, leading to a decrease in the decomposition temperature.

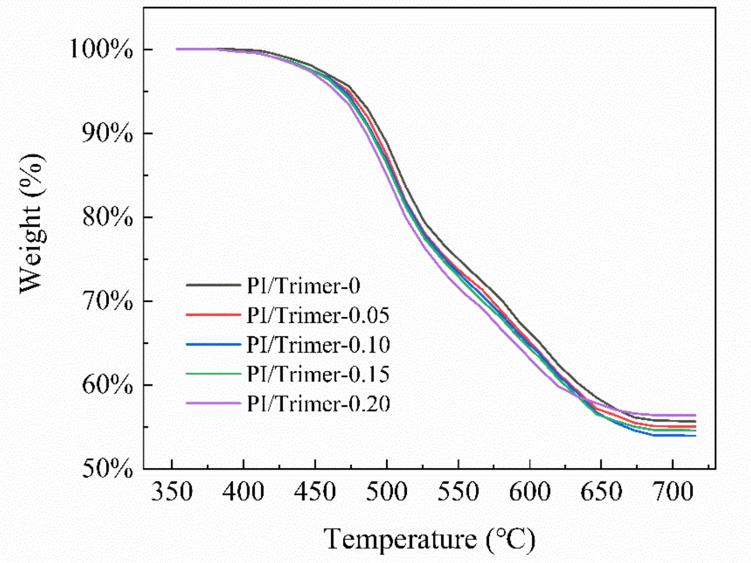

Figure S3. TGA curve of PI films modified with HDI trimer.
